# Nitrate of Silver in Epidemic Dysentery

**Published:** 1850-09

**Authors:** Lewis Slusser

**Affiliations:** Canal Fulton, Ohio


### ARTICLE IV.

*Nitrate of Silver in Epidemic Dysentery.*---By Lewis Slusser, M. D., of Canal Fulton, Ohio.

That diseases of an epidemic character are more difficult to manage---more intractable in their nature and treatment, than the sporadic form, is a principle in the practice of medicine that will not, I presume be denied.

During the summer of 1849, Dysentery prevailed in this section with unwonted virulence. In some neighborhoods the mortality attending its prevalence was so alarming, that with some practitioners it was regarded as but another form of Asiatic Cholera.

In very many cases, the ordinary remedies, such as we had been accustomed to prescribe in former years, and with satisfactory results, utterly failed. Neither mercurials, opiates, nor astringents, separately, or in varied combination, exercised any control over the symptoms, not even palliating them. The same may be said of Ipecac., Hope's Mixture, and counter-irritation. Nor had injections of Starch and Laudanum, Ice Water, or suppositories of solid Opium any effect in mitigating the tormina and the tenesmus. Dr. Young's Buttermilk treatment (vide Amer. Jour. of Med. Sci., 1842), proved advantageous in a few cases; in others, it undoubtedly aggravated the symptoms. Antiphlogistics were contra-indicated. Some cases, despite the most energetic treatment, would terminate fatally in less than forty-eight hours; others, prostrated from the excessive evacuations, fell into a typhoid condition, lingered a fortnight or more, and then died. In this latter condition it was, after having failed with those remedies hitherto regarded as orthodox, that I had recourse to Nitrate of Silver, a remedy first suggested, I believe, in this disease, by M. Trousseau. In determining upon this article, I was mainly influenced by the knowledge of its frequent exhibition in other enteric affections, both acute and chronic; and particularly by the ocular proof of its beneficial effects in Typhoid Fever, which prevailed among us the previous Spring. Regarding the pathological conditions of the two affections, as in many respects analagous, I felt justified in giving the remedy a trial. The results were very satisfactory; and I may add, that subsequent experience confirms the favorable opinion previously entertained.

I have not had any experience of its effects in the *first* stage of Dysentery. In what some authors very properly, as I conceive, designate the *second* stage—where the discharges give evidence of an ulcerated condition of the bowels, accompanied with Typhoid symptons---I regard Nitrate of Silver as *the* remedy to be preferred to any I have yet seen recommended. I will give particulars of a few cases from notes taken at the time.

The first case in which I exhibited it, was that of Mrs. T., æt. about 35; the mother of four children. I had treated her in the Spring, for "sore mouth peculiar to nursing women." In June, she had an attack of Cholera Morbus, which yielded upon the exhibition of our ordinary remedies. About the first of August, Dysentery made its appearance in her family. First her husband was attacked; he convalesced in a few days upon the Calomel and Opium treatment. Next herself. Resorting to the previously tried remedies already mentioned without any mitigation of symptoms, her condition soon became such, that I was satisfied unless some other course of treatment was speedily adopted the result could not be otherwise than fatal. At this stage, decided typhoid symptoms had supervened; countenance hippocratic; skin bedewed with a cold clammy sweat; eyes, sunken and lustreless; pulse weak and frequent; tongue dry, red and glazed; thirst ardent; bowels tympanitic; tormina and tenesmus almost incessant; fifteen and twenty discharges in as many hours, of a purulent, bloody, and lymphous character.

I determined upon the following prescription:

℞. Argent. Nitrat. Crys., gr. vj.

Pulv. Opii., ℈j.

Mucil. Gum Acac., q. s.

M. ft. pil. No. xij. One every two hours.

Her prostrated condition was such as to demand the free exhibition of stimulants, in order to sustain the faltering energies of life. Brandy and Arom. Spts. Ammon. were given *pro re nata*. At the same time, I ordered an injection every three hours, of gr. ij. Nitrate of Silver dissolved in ℥j. warm water, mixed with a gill of tepid milk. At the expiration of twenty-four hours from the adoption of this treatment, I found an evident amelioration of the distressing symptoms. The evacuations were less frequent, and there was a decided mitigation of the tormina and tenesmus. I was encouraged to repeat the prescription, but prolonged the time of giving the pills to three hours, and omitted the injections. The discharges soon after exhibited the characteristic dark appearance, the effects of the remedy, and contained less mucus. The symptoms gradually abated, the secretions became natural, and in a few days the patient was entirely out of danger.

The next case was that of a daughter of Mrs. T., æt. 10 years. She had been confined about a week; condition much the same as that of her mother. Ordered the same prescription, observing a differential proportion. The improvement, for the first twenty-four hours was not so marked as that of her mother; and observing a want of action about the surface, I concluded upon the following:—

℞. Nitrate Argent. Crys., grs. jiss.
Sulph. Morph., gr. j.
Vin. Ipecac., ℨj.
Aquæ Camph., ℥j.

M. A teaspoonful every two hours. At the same time ordered a warm bath. This had the desired effect. Free perspiration followed; the alimentary secretions improved; and in a short time she also recovered.

Few days after, saw Mr. M——, æt. about 45, in consultation with Dr. Donahu. He had been laboring under Dysentery some twelve days, and was much prostrated. Pulse 130; abdomen tympanitic, though not tender upon pressure; had discharged on the day previous a large quantity of fatty matter, having the consistence of healthy pus, but inodorous; tongue dry, and covered with a thick white coat; the papillæ prominent; sordes upon the teeth and gums; his whole surface covered with a fœtid clammy perspiration. He had had the full benefit of the Calomel and Opium treatment. Typhoid symptoms were present, and it was evident there was a decided downward tendency.

The treatment adopted in case first was decided upon, and the results were equally fortunate.

I deem it unnecessary to extend this article, by a detailed history of other cases, with like symptoms, treated by the same curative agent, and resulting alike satisfactorily. Sufficient, I think, has already been adduced to recommend the agent as one at least worthy a trial. I might mention that I suggested the remedy to several neighboring practitioners, and so far as I have heard, its administration in the conditions before specified, was attended with uniform success.

In obstinate Diarrhœa of infants, it has proven in my hands an excellent remedy. In advanced stages, where the prostration and emaciation is extreme, dejections frequent and watery, I have exhibited the following mixture with admirable results:—

℞. Argent. Nitrat. Crys.,

Sulph. Morph. *aa.*, grs. ij.

Gum Arab., ʒj.

Sacch. Alb., ʒij.

Aquæ., f. ℥ii..

Ft. mix. Teaspoonful every three hours, to a child three years old.---*Medical Examiner.*

